# Diabetic cardiomyopathy: pathophysiology and clinical features

**DOI:** 10.1007/s10741-012-9313-3

**Published:** 2012-03-28

**Authors:** Takayuki Miki, Satoshi Yuda, Hidemichi Kouzu, Tetsuji Miura

**Affiliations:** Division of Cardiology, Second Department of Internal Medicine, School of Medicine, Sapporo Medical University, South-1 West-16, Chuo-ku, Sapporo, 060-8543 Japan

**Keywords:** Diabetes mellitus, Heart failure, Pathophysiology, Infarct size, Signal transduction, Therapy

## Abstract

Since diabetic cardiomyopathy was first reported four decades ago, substantial information on its pathogenesis and clinical features has accumulated. In the heart, diabetes enhances fatty acid metabolism, suppresses glucose oxidation, and modifies intracellular signaling, leading to impairments in multiple steps of excitation–contraction coupling, inefficient energy production, and increased susceptibility to ischemia/reperfusion injury. Loss of normal microvessels and remodeling of the extracellular matrix are also involved in contractile dysfunction of diabetic hearts. Use of sensitive echocardiographic techniques (tissue Doppler imaging and strain rate imaging) and magnetic resonance spectroscopy enables detection of diabetic cardiomyopathy at an early stage, and a combination of the modalities allows differentiation of this type of cardiomyopathy from other organic heart diseases. Circumstantial evidence to date indicates that diabetic cardiomyopathy is a common but frequently unrecognized pathological process in asymptomatic diabetic patients. However, a strategy for prevention or treatment of diabetic cardiomyopathy to improve its prognosis has not yet been established. Here, we review both basic and clinical studies on diabetic cardiomyopathy and summarize problems remaining to be solved for improving management of this type of cardiomyopathy.

## Introduction

The number of patients with diabetes has been increasing worldwide in the past two decade, and these patients are predisposed to serious cardiovascular morbidity and mortality [1]. The impacts of diabetes on the development of atherosclerotic vascular diseases have been established, and results of recent clinical trials have indicated that not only hyperglycemia but also other risk factors need to be controlled for preventing atherosclerotic vascular events in diabetic patients [2, 3]. On the other hand, non-ischemic heart failure associated with diabetes has received much less attention than coronary and cerebral vascular events.

**Fig. 1:**
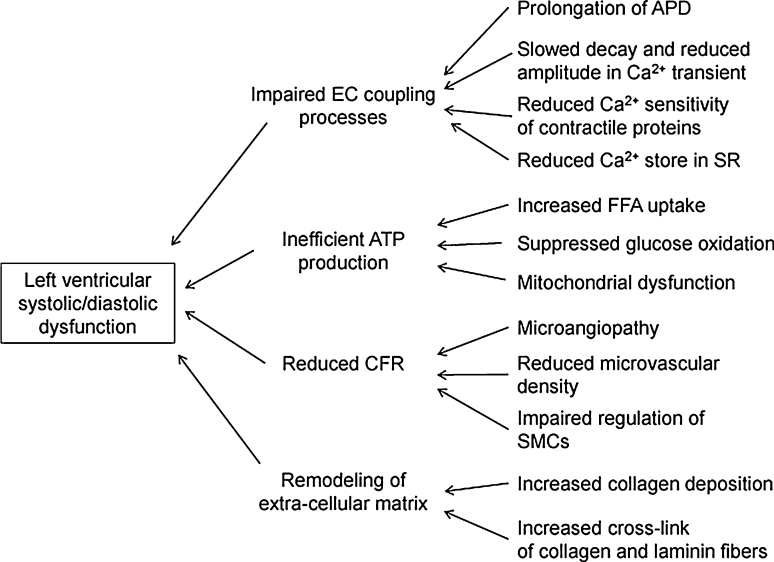
Proposed mechanisms of contractile dysfunction by diabetes. *EC coupling* excitation–contraction coupling, *APD* action potential duration, *SR* sarcoplasmic reticulum, *FFA* free fatty acid, *CFR* coronary flow reserve, *SMC* smooth muscle cell

**Fig. 2:**
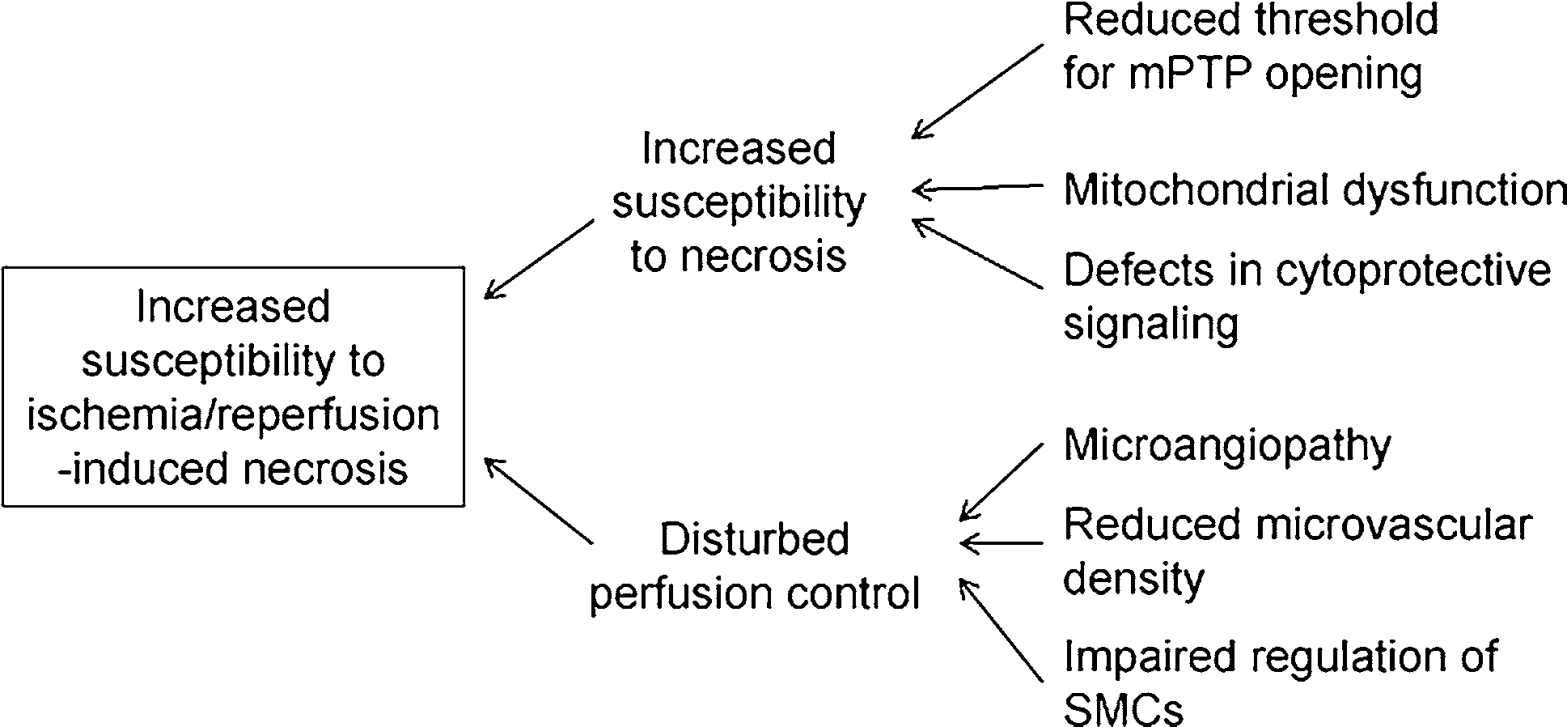
Proposed mechanisms of diabetes-induced increase in susceptibility of the myocardium to ischemia/reperfusion-induced infarction. *mPTP* mitochondrial permeability transition pore, *SMC* smooth muscle cell

Population-based studies have shown that the risk of heart failure is increased two- to threefold by diabetes [4, 5]. The presence of diabetes substantially accelerates development of heart failure in patients with myocardial infarction [6, 7], hypertension [8], or atrial fibrillation [9], leading to poorer prognosis. Diabetes predicts poor prognosis independently of coronary artery disease and level of left ventricular ejection fraction (LVEF) in heart failure patients [10, 11]. However, the concept of “diabetic cardiomyopathy” is still controversial, and a specific strategy to prevent or treat heart failure associated with diabetes has not been established. In this article, we review results from recent basic and clinical studies regarding “diabetic cardiomyopathy” and discuss its pathogenesis (Figs. 1, 2), diagnosis, and management. Although type 1 diabetes mellitus (T1DM) and type 2 diabetes mellitus (T2DM) differ in etiology and metabolic profiles, the two types share many features of cardiomyopathy. In this review, we mainly focus on myocardial changes that are commonly observed in both T1DM and T2DM and briefly discuss their differences if applicable.

## Definition and clinical phenotype of diabetic cardiomyopathy

### Definition of diabetic cardiomyopathy

“Diabetic cardiomyopathy” is a concept that was introduced in 1972 by Rubler et al. [12], who examined pathology of four autopsy cases with diabetic glomerulosclerosis and no known cause of heart failure. The current typical definition of diabetic cardiomyopathy comprises structural and functional abnormalities of the myocardium in diabetic patients without coronary artery disease or hypertension [13]. Obviously, however, this type of cardiomyopathy should be present also in diabetics with coronary artery disease and/or hypertension, though it is difficult to separately assess the contribution of diabetic cardiomyopathy to overall ventricular dysfunction in such cases.

Interstitial and perivascular fibrosis is a histological hallmark of diabetic cardiomyopathy [12, 14, 15], and the extent of fibrosis correlates with heart weight [15]. In addition to the increase in collagen deposition, cross-linking of collagen fibers may be increased by diabetes, contributing to reduction in ventricular compliance [16]. Clinical evidence to support this notion is actually sparse, but some studies [17–19] indicated that glycation of collagen fibers is indeed increased in hearts of diabetic patients.

“Cardiomyocyte hypertrophy” in diabetic cardiomyopathy is referred to in some earlier reviews, but its contribution to “ventricular hypertrophy” is not clear. Human myocardium biopsied at the time of coronary bypass surgery showed an increased cross-sectional area (CSA) of cardiomyocytes and interstitial fibrosis in diabetic patients compared with those in non-diabetics [20]. However, human biopsy studies by Yarom et al. [21] and Kawaguchi et al. [22] showed that the average myocyte diameter was not significantly increased by diabetes alone. Photographs of the histology of autopsy cases presented in earlier reports [12, 14, 15] show hypertrophic cardiomyocytes mixed with atrophic ones in diabetic cardiomyopathy. Increase in the CSA of cardiomyocytes with or without interstitial fibrosis has been reported for different animal models of T1DM and T2DM [23–25], but significant reduction in the CSA of cardiomyocyte was observed in a model of T1DM, Akita (*Ins* ^2WT/C96Y^) mouse [26]. Taken together, cardiomyocyte hypertrophy appears to be a frequently observed feature but not a requisite of diabetic cardiomyopathy. We speculate that long-standing metabolic derangements and modification of microcirculation (see section “Abnormalities in microvasculature” below) by diabetes induce different levels of hypertrophy, atrophy, and loss of cardiomyocytes in the myocardium depending on the duration of diabetes and/or co-morbidities such as hypertension.

Although it has not been included in the definition, increased susceptibility to ischemia/reperfusion injury may be an important feature of diabetic cardiomyopathy. Two clinical studies showed that myocardial infarct size after coronary reperfusion therapy was larger by 30–80 % in diabetic patients than in non-diabetic patients [27, 28]. The difference was observed even though coronary blood flow was similarly restored by percutaneous coronary intervention in the diabetic and non-diabetic groups [27].

### Clinical phenotype of diabetic cardiomyopathy

#### Ventricular morphology

Previous studies using transthoracic echocardiography (TTE) has indicated that diabetes (mostly T2DM) is associated with left ventricular (LV) hypertrophy or concentric LV remodeling (i.e., increased LV mass [LVM]-to-LV end-diastolic volume ratio) in females but not consistently in males [29–34]. However, TTE is not always suitable for elderly and/or obese patients, in whom image quality is frequently low. Magnetic resonance imaging (MRI) does not have such a disadvantage, and a recent study using this modality demonstrated significant association of insulin resistance and hyperglycemia with increase in LVM and LVM-to-LV end-diastolic volume ratio regardless of age and gender [35, 36]. Compared with studies on T2DM, few studies on T1DM have shown an increase in LV mass [37–42] possibly due to the younger age and lower incidence of hypertension in T1DM patients recruited to those studies.

Interstitial fibrosis in diabetic hearts can be assessed by integrated backscatter (myocardial ultrasound reflectivity) in two-dimensional echocardiography [43–45] and by late gadolinium (Gd) enhancement in cardiac MRI [46]. Two-dimensional echocardiography indicated an increased integrated backscatter index in the ventricular septum by 55 % and posterior wall by 15 % in diabetic patients as compared with that in non-diabetic controls [45]. Kwong et al. [46] reported that late Gd-enhancement in MRI was present in 28 % of diabetic patients without clinical evidence of myocardial infarction. Which of the two clinical methods is more sensitive for the detection of ventricular fibrosis in diabetic hearts remains unclear.

#### LV diastolic and systolic dysfunction

The most frequent echocardiographic finding in asymptomatic T1DM and T2DM patients is LV diastolic dysfunction with normal LVEF. Diastolic dysfunction is detectable in diabetic hearts without hypertrophy [37, 47, 48], indicating that hypertrophy is not a requisite of diabetes-induced ventricular dysfunction. It is difficult to rigorously characterize differences, if any, in ventricular dysfunction between T1DM and T2DM since age and co-morbidities in study subjects are not comparable between the studies on T2DM and those on T1DM.

**Fig. 3:**
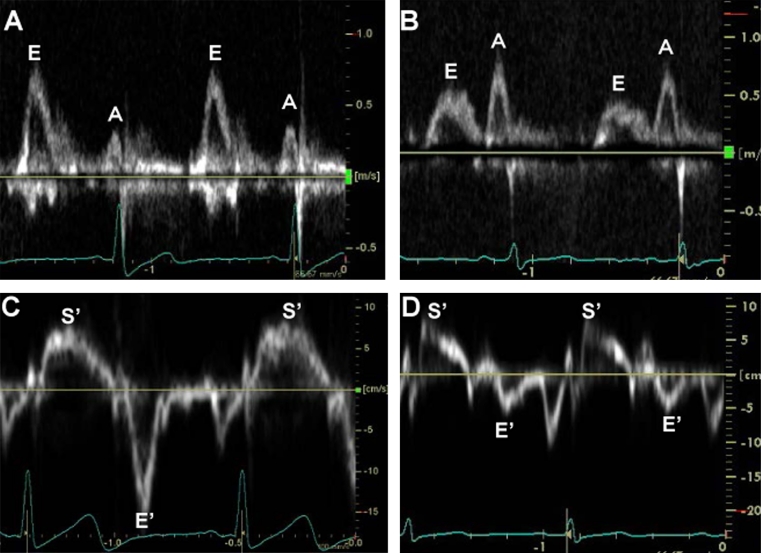
Examples of Doppler echocardiography in a healthy subject and a T2DM patient. Transmitral flow patterns are shown for a healthy subject (**a**) and a T2DM patient (**b**). Peak velocities during early diastole (*E*) and late diastole (*A*) are shown. *E*/*A* ratios are 2.2 and 0.6 in **a**, **b**, respectively. **c**, **d** show tissue Doppler imaging, with positioning of sample volume at the septal mitral annulus, in a healthy subject and a T2DM patient, respectively. The diabetic patient (**d**) had lower peak velocities during systole (*S*′) and early diastole (*E*′) (7.5 and 6.0 cm/s, respectively) than those in the healthy subject (**c** 8.5 and 15.0 cm/s, respectively)

LV diastolic dysfunction evaluated from transmitral LV filling pattern (i.e., abnormal relaxation and/or pseudonormal filling) (Fig. 3) was observed in 47–75 % of asymptomatic normotensive patients with well-controlled T2DM [49–51]. Tissue Doppler imaging (TDI) (Fig. 3) is more sensitive for detection of LV dysfunction than conventional TTE. It enables measurement of myocardial tissue velocities in the longitudinal direction, and the peak early diastolic myocardial velocity (*E*′) reflects the global LV diastolic function. Kosmala et al. [52] and Di Bonito et al. [53] reported that *E*′ was significantly lower in diabetic patients without hypertension than in normal subjects. In a study by Boyer et al. [51], TDI showed LV diastolic dysfunction in 63 % of asymptomatic T2DM patients, while conventional Doppler echocardiography showed the dysfunction in only 46 % of the subjects.

**Fig. 4:**
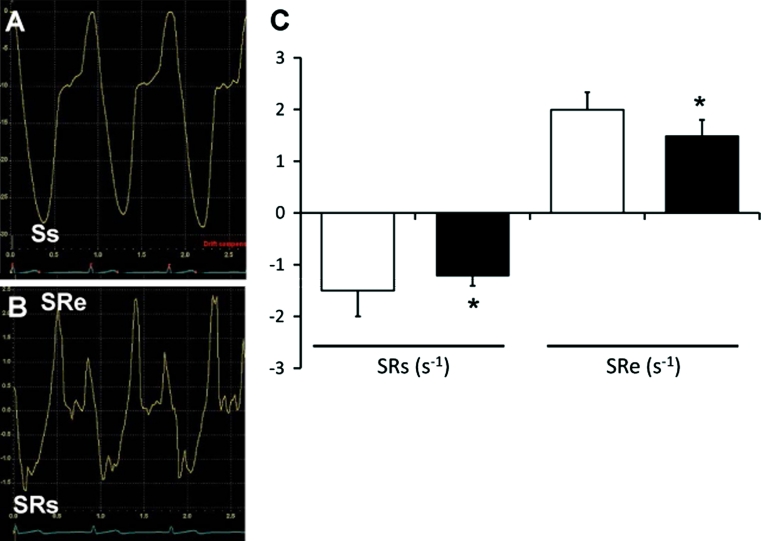
Tissue Doppler-derived strain and strain rate of the left ventricle. **a**, **b** show strain and strain rates, respectively, in a normal control. *Ss* septal peak strain, *SRs* strain rate in systole, *SRe* strain rate in early diastole. **c** shows comparison of LV strain rates in normal controls (*white bars*, *n* = 15) and normotensive T2DM patients without coronary artery disease (*black bars*, *n* = 15). *P < 0.05 versus control. (S. Yuda, unpublished data)

Systolic LV function is also impaired by diabetes, though its incidence appears lower than that of diastolic dysfunction. In several, but not all, studies in the literature, patients with diabetes mellitus had smaller LV fractional shortening (LVFS) and mid-wall shortening than those in subjects with normal glucose tolerance [30, 31, 35]. The discrepancy in the literature may be attributable to LV load dependence of LVFS and to relative insensitivity of LVFS in detecting subtle systolic dysfunction. In fact, more sensitive indices of systolic function in TDI and strain rate imaging (SRI) (Fig. 4) consistently indicate subclinical reduction in LV systolic function by diabetes [54–56].

Three studies using TDI [52, 54, 55] showed that the peak systolic velocity (*S*′) was 11–20 % lower in normotensive T2DM patients than in non-diabetic subjects, though LVEFs were similar. SRI enables quantitative measurement of regional LV function independent of cardiac rotational motion and tethering effect. Two-dimensional speckle tracking, by which strain rate in all three directions (longitudinal, circumferential and radial) can be determined without angle dependency, has been employed in recent studies for assessment of LV systolic and diastolic dysfunctions in T2DM patients [57, 58]. Ng et al. [57] reported that longitudinal strain was reduced with preserved radial/circumferential strains in asymptomatic patients with uncomplicated diabetes mellitus. More recently, Ernande et al. [58] showed that both longitudinal and radial strains were reduced after adjustment for blood pressure, age, and body mass index in asymptomatic diabetic patients. Significant LV dysfunction was also detected in T1DM by TDI [38, 40, 41]. Taken together, the findings by TDI and SRI suggest that impaired longitudinal LV shortening, reflecting subendocardial dysfunction, is one of earliest signs in diabetic cardiomyopathy.

#### Response to stress tests

Emerging evidence indicates the presence of latent LV dysfunction in diabetic hearts. Ha et al. [47] showed that *S*′ and *E*′ during an exercise test were significantly lower by 10–15 % in T2DM than in non-diabetic controls, though both *S*′ and *E*′ were within normal ranges at rest in the two groups. In a study by Jellis et al. [59], who defined abnormal *E*′ (septal *E*′) at rest as <2SD of normal for age and abnormal *E*′ at peak exercise stress as <−9.9 cm/s, *E*′ at stress was abnormally low in 49 % of the T2DM patients with normal *E*′ at rest. Palmieri et al. [60] reported that peak exercise stroke volume index and cardiac index were significantly lower in patients with uncomplicated T1DM than in non-diabetic normotensive controls, though LV TDI parameters were comparable in the two groups. Hence, it is likely that prevalence of diabetic cardiomyopathy is much higher than that previously thought in both types of diabetes. Furthermore, latent LV dysfunction caused by diabetes does not appear to be a trivial problem since blunted increase in systolic blood pressure/end-systolic LV volume ratio (SP/ESV) by exercise is associated with poor prognosis [61].

## Mechanism of contractile dysfunction of diabetic myocardium

Different animal models of T1DM (e.g., streptozotocin [STZ]-treated and alloxan-treated animals) and T2DM (e.g., Goto-Kakizaki rat, Otsuka-Long-Evans-Fatty rat [OLETF], ob/ob mouse) have been used for investigation of mechanisms by which diabetes impairs contractile function of the heart. In the following sections, we primarily discuss the findings commonly observed in both T1DM and T2DM models, unless otherwise stated.

### Impairments in excitation–contraction coupling

Diabetes significantly modifies action potential, Ca^2+^ transient and Ca^2+^ sensitivity of contractile elements in cardiomyocytes [62–66]. Prolongation of action potential duration (APD) and slower decay of Ca^2+^ transient are consistently observed in diabetic cardiomyocytes. It is notable that such changes in the Ca^2+^ transient were observed before development of systolic ventricular dysfunction. Peak amplitude of Ca^2+^ transient was reduced in some [64, 65, 67–69], but not all [70–72], models of diabetes.

As for prolongation of APD, reduction in transient outward K^+^ (Ito) current has been shown in most animal models of diabetes [62, 63, 65, 66, 73], though reduced expression of L-type Ca^2+^ channel was an additional abnormality in some models [64, 74]. The prolongation of APD is potentially a compensatory mechanism for preserving Ca^2+^ influx in cardiomyocytes with down-regulated L-type Ca^2+^ channel. However, it could lead to untoward outcomes. A study by Sah et al. [75] showed that down-regulation of Ito induces enhanced Ca^2+^ cycling and activation of calcineurin, leading to interstitial fibrosis and ventricular contractile dysfunction.

Down-regulation of Kv4.2 (one of alpha-subunit subfamilies of the voltage-gated K^+^ channel) expression underlies reduction in Ito current in diabetic hearts. The mechanism of the Kv4.2 down-regulation remains unclear, but inactivation of pyruvate dehydrogenase (PDH) and activation of peroxisome proliferator-activated receptor-α (PPARα) may be involved. In the diabetic myocardium, PDH is inhibited by PDH kinase-4 (PDK4) [76–78], and inhibition of PDH by 3-bromopyruvate has been shown to reduce the Ito current in normal cardiomyocytes [79]. Conversely, reduced Ito current in postinfarct remodeling hearts was restored by 4–5 h treatment with dicholoroacetate or pyruvate [79]. PDK4 is one of enzymes that are up-regulated by activation of PPARα [80, 81], and chronic cardio-specific activation of PPARα has been shown to down-regulate protein expression of both α-subunit (Kv4.2/KCND2) and β-subunit (KChIP2/KCNIP2) of the Ito channel, reducing Ito current density [82]. Hence, it is possible that PPARα activation by increased fatty acid uptake is upstream of PDH inhibition in the mechanism of Kv4.2 down-regulation by diabetes.

Slowed decay in Ca^2+^ transient in diabetic cardiomyocytes is theoretically attributable to reduced rate of Ca^2+^ removal from the cytosol and/or reduced affinity of troponin C, a major Ca^2+^ buffer in the cytosol, for Ca^2+^. As for the effect of diabetes on affinity of troponin C to Ca^2+^, a study by Ishikawa et al. [70], the only one to our knowledge, showed that Ca^2+^ affinity of troponin C was similar in STZ-induced diabetic and non-diabetic cardiomyocytes. On the other hand, studies using different animal models of diabetes consistently indicated reduction in protein level of sarcoplasmic reticulum Ca^2+^ ATPase 2a (SERCA2a) [83], and phospholamban phosphorylation was enhanced in some of the models [63, 64]. In addition, posttranslational modifications of SERCA2a by diabetes were reported by Bidasee et al. [84]; they found non-enzymatic glycosylation of SERCA2a (i.e., formation of advanced glycation end products [AGEs] on SERCA—see section “Remodeling of extracellular matrix” for details) in a model of T1DM, which potentially compromises pump activity of SERCA2a. Another Ca^2+^ handling protein for Ca^2+^ efflux, Na^+^–Ca^2+^ exchanger (NCX), is preserved in the diabetic heart [71]. These findings indicate that down-regulation of SERCA2a is a primary mechanism of delayed decay in Ca^2+^ transient. The mechanism by which diabetes reduces SERCA2a expression is unclear, though involvement of nuclear *O*-GlcNAcylation was recently suggested by results of experiments using adenovirus-mediated overexpression of *O*-GlcNac transferase and *O*-GlcNAcase [85, 86].

Increased leakage of Ca^2+^ from the sarcoplasmitc reticulum (SR) has also been reported as an abnormality in diabetic hearts. Belke et al. [68] showed that Ca^2+^ leak under blockades of NCX and ryanodine receptors (RYRs) was significantly increased in ob/ob mice and that the increase was associated with reduced expression of FKBP12.6, a regulatory factor of RYRs. In a study that determined local SR Ca^2+^ release as “Ca^2+^ sparks” by use of a fluorescent Ca^2+^ probe, frequency of the Ca^2+^ sparks was increased by 60 % in association with reduction of both RYR2 and FKBP12.6 by 50 % in the myocardium of rats with STZ-induced diabetes [87]. Interestingly, the increase in local Ca^2+^ sparks and down-regulation of RYR2 and FKBP12.6 were attenuated by candesartan, indicating involvement of AT_1_ receptor activation [69]. The Ca^2+^ leak via dysfunctional RYRs and reduced Ca^2+^ uptake by SERCA2a appear to be responsible for significant reduction in SR Ca^2+^ store by diabetes [67].

Change in Ca^2+^ sensitivity of contractile proteins by diabetes is controversial. Data are contradictory (i.e., decrease vs. increase in Ca^2+^ sensitivity) even in the same T1DM model (STZ-induced diabetes) [88, 89]. In a study using cardiomyoytes from T2DM patients undergoing coronary artery bypass surgery, significant reduction of Ca^2+^ sensitivity was observed [90]. There is no clear explanation for the contradictory results.

### Metabolic derangements

Turnover of ATP (up to 35 kg/day) is many times of its pool, and extraction of energy from substrates is not very large (~25 %) in the myocardium [91–93]. Thus, a small reduction in the efficiency of ATP synthesis could significantly compromise cellular functions, including contraction and relaxation. Diabetes reduces the efficiency of energy production by increase in fatty acid uptake and suppression of glucose oxidation. Fatty acid oxidation is augmented not only by elevation of plasma level of fatty acid but also by activation of PPARα. PPARα is activated by intracellular fatty acids and up-regulates multiple enzymes relevant to fatty acid metabolism [94, 95]. PPARα contributes also to suppression of glucose oxidation by up-regulation of PDK4 transcription [96]. Most of the cytosolic long-chain acyl-CoAs are used for β-oxidation in mitochondria, and approximately 80 % of acetyl-CoA in the heart of a model of T2DM, db/db mouse, was found to be fatty acid-derived both when perfused with low glucose/low fatty acid buffer and when perfused with high glucose/high fatty acid buffer [97].

Glucose oxidation is inhibited at multiple steps in diabetic hearts. Uptake of glucose is impaired in diabetic hearts by down-regulated expression of GLUT4/GLUT1 and by blunted sarcolemmal translocation of GLUT4 in response to insulin [98, 99]. Impaired tyrosine phosphorylation of the insulin receptor and insulin receptor substrates and blunted activation of PI3K-Akt signaling are involved in the deficient response of diabetic hearts to insulin. In addition, activities of hexokinase, phosphofructokinase, and PDH are inhibited by long-chain acyl-CoA, citrate, and PDK4 in the diabetic myocardium [96, 100, 101].

Mitochondrial dysfunction is also responsible for reduced efficiency in energy production in the diabetic heart as recently reviewed by Bugger and Able [102]. Production of cytotoxic reactive oxygen species (ROS) was augmented in mitochondria in different types of diabetes. Increased fatty acid oxidation, which has higher oxygen cost than glucose, and increased activity of uncoupling proteins (UCPs) in mitochondria appear to underlie the augmented ROS production. ROS can directly activate UCP3 and further reduce efficiency of ATP production in mitochondria [103, 104].

Extra-mitochondrial ROS level is also increased in a model of T2DM (obese Zucker rat). In this model, increased metabolic flux to the pentose phosphate pathway augments generation of Nox-derived ROS by the elevation of NADPH level due to up-regulated activity of glucose-6-phophate dehydrogenase (G6PD) [105]. Protein kinase C (PKC) contributes to the up-regulation of G6PD. However, such up-regulation of G6PD activity in the myocardium was not detected in a model of T1DM (STZ-induced diabetes) [106].

An important question is whether supply of ATP is indeed insufficient for its demand in diabetic cardiomyocytes. A clue to the answer to this question is change in the phosphocreatine (PCr)/ATP ratio. Reduction of PCr/ATP ratio indicates suppressed ATP production and/or suppressed production of PCr from ATP by the creatine kinase (CK) system. Determination of PCr and ATP in the human myocardium by magnetic resonance spectroscopy (MRS) showed that the PCr/ATP ratio is significantly reduced by diabetes and that the ratio negatively correlates with plasma free fatty acid level or live triglyceride level [107, 108]. These observations support the notion that supply of ATP in response to intracellular demand is compromised in diabetic hearts. It is notable that the cardiac metabolic derangement indicated by PCr/ATP precedes ventricular dysfunction detectable at rest in the diabetic heart but possibly underlies the dysfunction unmasked by stress tests (section “Response to stress tests”).

### Remodeling of extracellular matrix

Distinguishing from enzymatic glycosylation of proteins, non-enzymatic formation of stable glycosylation product by Amadori rearrangement (Amadori product) is called glycation. Glycated proteins undergo a series of chemical rearrangements to form complex compounds with cross-links, which are referred to as advanced glycation end products (AGEs). AGEs have been shown to be increased in plasma by hyperglycemia, aging, and renal failure [16, 109–111]. Accumulation of AGE in collagen was associated with reduced collagen turnover, indicating the possibility that cross-linking of collagen makes collagen resistant to hydrolytic turnover [112]. Such AGE-mediated cross-linking of collagen is thought to be responsible for increased stiffness of arteries and the myocardium. In fact, AGE in the myocardium increases in T1DM and T2DM, and positive correlations of serum level of AGEs with ventricular isovolumetric relaxation time, arterial stiffness, and carotid intimal thickness have been shown in diabetics [109, 110, 113]. Furthermore, treatment with an inhibitor of AGE formation (aminoguanidine) prevented ventricular dysfunction in diabetic rats [114, 115]. Treatment with alagebrium (ALT-711), an “AGE cross-link breaker”, restored the LV function and reduced myocardial collagens in a canine model of diabetes [116], and its beneficial effect on LV diastolic function was suggested in heart failure patients with preserved ejection fraction [117].

Fibrosis in the cardiac interstitium and perivascular space in diabetic patients is reproducible in animal models at the late stage of diabetes. Contribution of the AT1 receptor to fibrosis is supported in the models of diabetes by three lines of evidence. AT1 receptor activity was up-regulated in diabetic hearts [118, 119], and this receptor is coupled with transforming growth factor-β1 (TGF-β1) signaling, which stimulates collagen production [120, 121]. Inhibition of AT1 receptor activity by AT1 receptor blockers (ARBs) or angiotensin converting enzyme (ACE) inhibitors ameliorated interstitial fibrosis and significantly improved LV function [120]. It should be noted that extent of interstitial fibrosis and that of glycation of proteins do not necessarily change in parallel [89].

### Abnormalities in microvasculature

“Microangiopathy” has been demonstrated in the myocardium of diabetic patients, and it was reproducible in a rat model of diabetes [12, 14, 21, 22, 122, 123]. Thickening of the capillary basement membrane, medial thickening of the arteriole, and perivascular fibrosis were observed in autopsy samples of the ventricular myocardium by conventional histology [12, 14, 21, 22]. Visualization of three-dimensional morphology of microvessels by use of the microfill technique showed microaneurysms, spasm, and spiral deformation of microvessels in the myocardium of T1DM and T2DM [122]. These vascular changes are reproducible in rat hearts by STZ-induced diabetes and hypertension [123].

In addition to morphology, density of the microvessels is modified in the heart by diabetes. Yoon et al. [124] showed that expression of vascular endothelial cell growth factor (VEGF) in the heart is down-regulated by diabetes and that the down-regulation is closely associated with reduction in capillary density, apoptosis of endothelial cells and interstitial fibrosis. Since insulin induces VEGF expression via PI3K-Akt signaling [125], impairment of this signaling in the diabetic heart may be responsible for the down-regulation of VEGF expression. Furthermore, restoration of VEGF by intramyocardial injection of plasmid DNA encoding VEGF prevented loss of capillaries in diabetic mice [124]. Unfortunately, data on VEGF expression in the human diabetic myocardium are contradictory in the literature [126–128]. VEGF mRNA level in ventricular biopsy samples from diabetic patients was reportedly reduced [126], not changed in the non-ischemic area and reduced in the ischemic area [127] or increased [128] as compared with non-diabetic patients. Difference between clinical backgrounds in study subjects might be involved in the contradictory results in human studies.

Reduction in coronary blood flow reserve (CFR) by diabetes has been demonstrated in both clinical and experimental studies [129–131]. In diabetic patients, CFR was inversely correlated with an index of LV relaxation (time from R-wave on the electrocardiogram to the onset of relaxation) [131]. In a rat model of obese T2DM (OLETF), CFR was reduced and inversely correlated with wall-to-lumen ratio of arterioles (<100 μm in diameter) and with extent of perivascular fibrosis [129]. Activation of the receptor for AGE (RAGE) in the endothelium by AGE inhibits production of nitric oxide (NO) and up-regulates expression of cell adhesion molecules [16]. Use of an AGE cross-link breaker, alagebrium, significantly improved flow-mediated dilatation in hypertensive patients [132]. Taken together, blunted NO production, AGE-mediated stiffening of coronary media, reduced angiogenesis, and perivascular fibrosis are possibly responsible for the reduction of CFR in diabetic hearts.

## Myocardial tolerance against ischemia/reperfusion-induced necrosis

### Changes in myocardial susceptibility to infarction by diabetes

Although clinical studies indicate enlargement of infarct size by diabetes in patients treated with reperfusion therapy [27, 28], animal studies have shown different diabetes-induced changes in infarct size as summarized in Table 1 [119, 133–181]. There were multiple differences in the experimental preparations and protocols, and a single factor cannot explain the discrepancy in effects of diabetes on infarct size. However, duration of the diabetic state and plasma level of insulin (i.e., T1DM vs. T2DM) appear to influence myocardial tolerance against infarction. In a study by Ravingerová et al. [147], infarct size after 30-min ischemia was smaller in diabetic rat hearts at 1 week after STZ injection than in controls, but this infarct size limitation was not detected 8 weeks later. Two other studies have also shown that increased resistance of diabetic hearts to ischemia/reperfusion injury at the early phase of diabetes later disappeared [149, 150]. However, enlargement of infarct size as early as 8 days after STZ injection was also reported [134, 135, 137], indicating involvement of a factor other than diabetes duration in infarct size change. As for insulin level, diabetic models with obesity and hyperinsulinemia [119, 140, 142–144], except for a few reports [153, 165], showed larger infarct size than that in non-diabetic controls.

**Table 1:** Effects of diabetes and hyperglycemia on infarct size

Diabetes
Infarct size enlargement
STZ (±alloxan)-induced DM (dog, rat, mouse): Refs. [133–139]
Zucker rat: Refs. [140–142]
OLETF rat: Refs. [119, 143]
ob/ob mouse: Ref. [144]
Infarct size reduction
STZ (±alloxan)-induced DM (rabbit, rat): Refs. [145–152]
Zucker rat: Ref. [153]
GK rat: Refs. [153, 154]
No change in infarct size
STZ (±alloxan)-induced DM (dog, rabbit, rat, mouse): Refs. [147, 149, 150, 155–165]
GK rat: Refs. [166–169]
ob/ob mice: Ref. [165]
Hyperglycemia
Infarct size enlargement
Dextrose or glucose i.v. infusion (dog, rabbit, rat): Refs. [170, 171, 173]
No change in infarct size
Dextrose or glucose i.v. infusion (dog, rabbit, rat): Refs. [158, 170, 172, 174–178]
Metabolic syndrome
Infarct size enlargement
Rat (Western diet): Ref. [179]
No change in infarct size
Rat (high fat diet, WOKW rat): Refs. [180, 181]

*GK rat* Goto-Kakizaki rat, *OLETF rat* Otsuka Long-Evans-Tokushima Fatty rat, *STZ* streptozotocin, *WOKW rat* Wistar-Ottawa-Karlsburg W rat

### Diabetes-induced defects in intracellular protective signaling

Diabetes is one of the pathological states that impair intracellular signaling for cardiomyocyte protection. Except for a few studies, previous studies showed that cardioprotection achieved by ischemic preconditioning (IPC) or ischemic postconditioning (IPost) is lost or required extra-cycles of “conditioning” in experimental diabetes (Table 2) [141, 144, 145, 151, 153, 157, 159, 160, 163, 165, 166, 182]. Mimetics of IPC and IPost (diazoxide, erythropoietin, [D-Ala^2^, D-Leu^5^]-enkephalin acetate [DADLE] and isoflurane) were also ineffective in limitation of infarct size in diabetic hearts [119, 141, 143, 158, 161–163, 169], confirming impairment of protective signaling by diabetes.

**Table 2:** Effects of diabetes and hyperglycemia on cardioprotection afforded by pre- and postconditioning and their mimetics

Diabetes
Preserved protection
Ischemic PC (rat): Refs. [145, 166]
GSK-3β inhibitors (rat): Refs. [119, 143, 161, 162, 182]
PDE 3 inhibitor (rat): Ref. [169]
PPAR-α agonist (rat): Ref. [168]
Metformin (rat): Ref. [167]
Impaired protection
Ischemic PC (dog, rabbit, rat): Refs. [141, 151, 153, 157, 159, 166, 182]
Ischemic PC (SWOP) (rabbit): Ref. [160]
Ischemic PostC (rat, mouce): Refs. [144, 163, 165]
Erythropoietin (rat): Refs. [119, 143, 162]
K_ATP_ channel opener (dog, rat): Refs. [141, 158]
Opioid agonists (rat): Refs. [119, 161]
Volatile anesthetics (rat): Refs. [163, 169]
Hyperglycemia
Preserved protection
Volatile anesthetics (dog, rat): Refs. [172, 176]
Impaired protection
Ischemic PC (dog): Refs. [170, 175]
K_ATP_ channel opener (dog): Ref. [158]
Volatile anesthetics (dog, rabbit, rat): Refs. [172, 174, 176–178]
Metabolic syndrome
Impaired protection
Ischemic PostC (rat): Ref. [181]

*PC* preconditioning, *PostC* postconditioning, *GSK*-*3β* glycogen synthase kinase-3β, *K*_*ATP*_ channel ATP-sensitive potassium channel, *PDE3* phosphodiesterase 3, *PPAR*-*α* peroxisome proliferator-activated receptor-α, *SWOP* second window of protection

Like animal models of diabetes, diabetic human hearts have defects in cytoprotective mechanisms. Ishihara et al. [183] showed that preinfarct angina pectoris, a clinical counterpart of IPC, reduced CK release and improved recovery of cardiac function and in-hospital survival after acute myocardial infarction in non-diabetic patients but not in diabetics. Impairment of IPC in human diabetes was also shown during angioplasty [184] and during a treadmill exercise test [185] by use of electrocardiographic severity of ischemia as an endpoint. Direct evidence for diabetes-induced loss of IPC protection in human hearts was provided by an in vitro experiment using atrial trabeculae obtained at open heart surgery. IPC failed to suppress CK release and contractile dysfunction after hypoxia/reoxygenation in vitro in atrial trabeculae from diabetic patients [186, 187].

Multiple defects in cytoprotective signal pathways have been indicated in diabetic hearts. Our recent studies have shown that Jak2, being upstream of PI3K-Akt signaling, is inhibited by enhanced calcineurin activity and that phosphorylation of GSK-3β by ERK is lost by an endoplasmic reticulum stress-dependent mechanism in a rat model of T2DM [119, 143]. Furthermore, protein level of active GSK-3β, a pro-necrotic and pro-apoptotic kinase, was increased in mitochondria, leading to increase in susceptibility of mitochondrial permeability transition in response to calcium overload [143]. On the other hand, a protective mechanism downstream of GSK-3β phosphorylation appears to be intact in diabetic hearts, since direct inhibitors of GSK-3β limit infarct size similarly in diabetic and non-diabetic animals [119, 143, 161, 162, 182].

There is limited information on whether glycemia control repairs defects in protective signaling in diabetic hearts. Acute hyperglycemia induced by dextrose infusion impaired infarct size limitation by IPC, a mitochondrial K_ATP_ channel opener and anesthetic agents [158, 170, 172, 174–178], indicating a primary role of hyperglycemia in impairment of protective signaling. Recently, Przyklenk et al. [165] reported that the cardioprotective effect of IPost was re-established in STZ-induced diabetic mice by pancreas islet cell transplantation. Transplantation of islet cells in diabetic mice normalized blood glucose level and also ERK signaling activated by IPost. Since dyslipidemia reportedly attenuates the infarct size-limiting effect of IPC [188–190], restoration of the protective effect of IPost in the diabetic heart by islet cell transplantation could have been a result of normalization of both plasma glucose and lipid profile. Nevertheless, circumstantial evidence to date supports the notion that normalization of the metabolic profile restores protective signaling mechanisms in the diabetic heart.

## Clinical diagnosis of diabetic cardiomyopathy

Currently, the best approach to the diagnosis of diabetic cardiomyopathy is detection of functional and structural changes in the LV and exclusion of other heart diseases being responsible for the changes in a diabetic patient. Diagnostic clues of diabetic cardiomyopathy are listed in Table 3, TDI and SRI being the most practical for detection of diabetic cardiomyopathy on a daily basis.

**Table 3:** Diagnostic clues of diabetic cardiomyopathy

Structural changes
LV hypertrophy assessed by 2D echocardiography or CMR
Increased integrated backscatter in the LV (septal and posterior wall)
Late Gd-enhancement of the myocardium in CMR
Functional changes
LV diastolic dysfunction assessed by pulsed Doppler echocardiography and TDI
LV systolic dysfunction demonstrated by TDI/SRI
Limited systolic and/or diastolic functional reserve assessed by exercise TDI
Metabolic changes
Reduced cardiac PCr/ATP detected by ^31^P-MRS
Elevated myocardial triglyceride content detected by ^1^H-MRS

*CMR* cardiac magnetic resonance imaging, *2D* two dimensional, *LV* left ventricular, *MRS* magnetic resonance spectroscopy, *SRI* strain/strain rate imaging, *TDI* tissue Doppler imaging

Left ventricular diastolic dysfunction detectable by TDI (and possible also by SRI) at exercise stress may be the earliest sign of diabetes-induced LV dysfunction as discussed in section “Clinical phenotype of diabetic cardiomyopathy”. Thus, normal echocardiographic findings at rest do not exclude presence of diabetic cardiomyopathy. Studies to date support the notion that diastolic dysfunction develops earlier than systolic dysfunction in diabetic hearts. However, Ernande et al. [191] recently reported that systolic longitudinal strain rate was abnormal in 28 % of diabetic patients with normal diastolic function and in 35 % of those with diastolic dysfunction. Assessment of interstitial fibrosis by integrated backscatter or Gd-enhancement of cardiac MRI is possible [43–46], but its diagnostic value has not yet been established.

A promising novel approach to diagnosis of diabetic myopathy is characterization of metabolic changes in the myocardium by ^31^P-MRS and by^1^H-MRS. As discussed in section “Metabolic derangements”, the PCr/ATP ratio, an index of energy charge, is reduced in the myocardium of diabetic patients compared with that in control subjects. Recent studies using ^1^H-MRS have demonstrated that increase in myocardial triglyceride content (i.e., myocardial steatosis) was associated with LV diastolic dysfunction in diabetic patients [192, 193]. Furthermore, Ng et al. [194] showed that myocardial steatosis was independently correlated with LV longitudinal strain and with systolic and diastolic strain rates determined by two-dimensional speckle tracking imaging in patients with uncomplicated diabetes mellitus. The possibility that myocardial steatosis is a specific marker of the diabetic cardiomyopathy warrants further investigation.

## Prevention and treatment of diabetic cardiomyopathy

### Prevention of diabetic cardiomyopathy

Although high prevalence of subclinical myocardial dysfunction has been reported in the early stage of T1DM, clinically relevant heart failure is relatively rare in this type of diabetes. In an observational study, 462 T1DM patients without a previous history of heart disease were followed up, and it found that only 17 patients (3.7 %) developed heart failure during a 12-year follow-up period [195]. The patients who developed heart failure in this cohort were older and had longer diabetes durations (35 ± 9 years), higher blood pressure, and higher prevalence of albuminuria and retinopathy than those in patients without heart failure. In contrast, heart failure develops more frequently in patients with T2DM [4, 5], which is frequently associated with other co-morbidities, such as hypertension, predisposing to heart failure. Hence, it is unlikely that glycemic control alone is sufficient for the prevention of diabetic cardiomyopathy.

A number of clinical trials have been conducted to evaluate the impact of glycemic control on the prevention of cardiovascular events in T2DM. However, end-points in the studies were atherosclerotic cardiovascular events and death, leaving non-ischemic heart failure not specifically determined. A recently published meta-analysis including a total of 27,049 subjects in the UKPDS 33 (UK Prospective Diabetes Study 33), ACCORD, ADVANCE, and VADT trials showed that mortality was not affected by intensive glycemic control, with hazard risks of 1.10 for cardiovascular death (95 % confidence interval [CI]; 0.84–1.42) and 1.04 for all-cause death (95 % CI: 0.90–1.20) [196]. These findings may appear to argue against the notion that tight glycemic control is beneficial for prevention of diabetic cardiomyopathy. However, the results do not preclude the possibility that intensive glycemic control commenced at an earlier stage of diabetes together with control of other risk factors prevents heart failure in diabetic patients. This speculation is supported by a few lines, at least, of evidence. First, clinical studies using TDI showed that glycemic control improved LV diastolic function in T2DM [197, 198]. Second, the Steno-2 trial [199] showed that simultaneous control of glycemia, hypertension, and dyslipidemia significantly reduced cardiovascular events and mortality in T2DM patients. Third, a recent meta-analysis of clinical trials on hypertension indicates that diabetes increases incidence of heart failure by more than fourfold in hypertensive patients [8].

Whether incidence and/or outcome of heart failure differ depending on the type of hypoglycemic agent selected for hyperglycemia control remains unclear. This issue has not been addressed by a prospective randomized clinical trial. In observational cohort studies and retrospective analyses of registered patients, use of metformin is associated with low incidence of heart failure compared with other glycemia control regimens [200]. Furthermore, clinical outcomes in diabetic patients with heart failure were better in groups treated with metformin [201, 202]. Aguilar et al. [202] matched metformin-treated and metformin-untreated groups of diabetic patients with heart failure and showed that mortality was lower in the metformin-treated group.

In contrast with metformin, thiazolidinedione (TZD) has been shown to increase incidence of “heart failure” in diabetes compared with sulfonylurea. Unfortunately, it is not clear whether the increase in “heart failure” by TZD indeed reflects worsening of LV function or just retention of fluids [203–206]. In fact, recent studies have suggested a favorable effect of TZD on cardiac function [207, 208]. Six months of treatment with pioglitazone improved diastolic function assessed by Doppler echocardiography in hypertensive patients in proportion to the amelioration of insulin resistance [207]. The same duration of treatment with pioglitazone was also reported to improve diastolic function and LV compliance assessed by MRI in uncomplicated T2DM patients [208]. It is notable, however, that improvement in the function could not be explained by treatment-related myocardial metabolic change assessed by positron emission tomography and MRS. Nevertheless, prospective clinical trials are necessary to clarify efficacies of hypoglycemic agents in prevention of diabetic cardiomyopathy.

### Management of heart failure in diabetic patients

Optimal treatment of heart failure in diabetic patients has not been specifically addressed, and relevant information is limited to effects of diabetes on the efficacy of a heart failure therapy in subgroup analyses of trials. In earlier studies, ACE inhibitor was suggested to be similarly effective in diabetic and non-diabetic patients with heart failure with reduced LVEF [209, 210] and that was the case for ARB as well [10]. In contrast, the benefit of a β-blocker on mortality may be attenuated in diabetic patients, especially in elderly patients [211, 212]. Under the standard treatment with these agents, prognosis of heart failure patients with diabetes is worse than that of heart failure patients without diabetes irrespective of LVEF levels [10, 213]. Heart failure with preserved LVEF is a primary phenotype in diabetes, and therapy to improve prognosis of this type of heart failure in general is still under intensive investigation [214].

## Perspectives

Accumulating evidence obtained by novel imaging techniques (i.e., TDI, SRI, MRS) indicates that these techniques for functional and metabolic analyses of human hearts will make it possible to formulate clinical parameters for diagnosis of diabetic cardiomyopathy. Such diagnostic criteria would facilitate the design of prospective studies to search for optimal therapy for prevention and treatment of this cardiomyopathy. Numerous questions regarding pathogenesis of diabetic cardiomyopathy still remain, but molecular mechanisms of down-regulation of SERCA2a, mitochondrial dysfunction, and defects in cytoprotective signaling appear particularly important issues for designing novel therapies for restoration of contractile function and prevention of progressive heart failure. Novel therapy is in urgent need since even mild diastolic dysfunction in diabetic hearts has been shown to be associated with more than a threefold increase in all-cause mortality [215].
